# Development and validation of a shoulder-specific body-perception questionnaire in people with persistent shoulder pain

**DOI:** 10.1186/s12891-021-03944-z

**Published:** 2021-01-21

**Authors:** Tomohiko Nishigami, Akihisa Watanabe, Toshiki Maitani, Hayato Shigetoh, Akira Mibu, Benedict Martin Wand, Mark J. Catley, Tasha R. Stanton, G. Lorimer Moseley

**Affiliations:** 1 Department of Physical Therapy, Faculty of Health and Welfare, Prefectural University of Hiroshima, 1-1, Gakuen-chou, Mihara, Hiroshima, 723-0053 Japan; 2 Department of Rehabilitation, Machida Orthopaedics, Kochi, Japan; 3 Department of Rehabilitation, Utsumi Orthopaedics Clinic, Kagawa, Japan; 4 Department of Rehabilitation, Miura internal Medicine Michiko Pediatrics Clinic, Kagawa, Japan; 5 Department of Nursing and Physical Therapy, Konan Woman’s University, Kobe, Hyogo Japan; 6 The School of Physiotherapy, The University of Notre Dame Australia, Fremantle, WA Australia; 7 Innovation, Implementation and Clinical Translation in Health (IIMPACT in Health), University of South Australia, Adelaide, SA Australia; 8 Neuroscience Research Australia, Sydney, NSW Australia

**Keywords:** Shoulder pain, Body perception, Rasch analysis, Reliability, Validity

## Abstract

**Background:**

There is evidence that people with persistent shoulder pain exhibit findings consistent with the presence of sensorimotor dysfunction. Sensorimotor impairments can manifest in a variety of ways, and further developing our understanding of sensorimotor dysfunction in shoulder pain may improve current models of care. The Fremantle Back Awareness Questionnaire (FreBAQ) has been developed to assess disturbed body perception specific to the back. The purpose of the present study was to develop a shoulder-specific self-perception questionnaire and evaluate the questionnaire in people with persistent shoulder pain.

**Methods:**

The Fremantle Shoulder Awareness Questionnaire (FreSHAQ-J) was developed by modifying the FreBAQ. One hundred and twelve consecutive people with persistent shoulder pain completed the FreSHAQ-J. Thirty participants completed the FreSHAQ-J again two-weeks later to assess test-retest reliability. Rasch analysis was used to assess the psychometric properties of the FreSHAQ-J. Associations between FreSHAQ-J total score and clinical status was explored using correlational analysis.

**Results:**

The FreSHAQ-J has acceptable category order, unidimensionality, no misfitting items, and excellent test-retest reliability. The FreSHAQ-J was moderately correlated with disability and pain catastrophization.

**Conclusions:**

The FreSHAQ-J fits the Rasch measurement model well and is suitable for use with people with shoulder pain. Given the relationship between the FreSHAQ-J score and clinical status, change in body perception may be worth assessing when managing patients with shoulder pain.

## Background

Shoulder pain is the third most common musculoskeletal disorder [1]. Between 7 and 34% of adults experience shoulder pain at any one time [2], and only half of new episodes of shoulder pain completely recover within the first six-months [3, 4]. Shoulder pain can lead to functional limitations and negatively impacts health-related quality of life [5, 6].

Data suggest that sensorimotor dysfunction could play an important role in shoulder pain [7–10]. People with persistent shoulder pain exhibit findings consistent with the presence of central sensitization [11], and neurophysiology studies have shown alteration in motor cortex function in people with shoulder problems [12–14]. Proprioceptive impairments have been demonstrated in people with rotator cuff pathology and with shoulder instability [15–18]. Furthermore, in healthy individuals, tactile acuity and motor imagery performance were associated with the physical performance of the shoulder [19]. Most importantly, studies have shown that interventions targeting sensorimotor function, such as motor control retraining exercises [20], proprioceptive exercise [21, 22], mirror therapy [23], and a combination of pain neuroscience education, tactile discrimination, and graded motor imagery [24] may increase function and reduce shoulder pain intensity.

Sensorimotor impairments can manifest in a variety of ways and thus further developing our understanding of sensorimotor dysfunction in shoulder pain may improve current models of care. One manifestation of sensorimotor dysfunction is disturbed body perception. This is a complex phenomenon that is attributed to multiple factors; as Pazzaglia and Zantedeschi elegantly describe [25]: *“knowledge of the body is filtered by perceptual information, recalibrated through predominantly innate stored information, and neurally mediated by direct sensory-motor information.”* Evaluation of disturbed self-perception therefore represents a way of capturing the conscious correlate of disruption across a range of sensorimotor processes. In addition, as pain may be viewed as a reflection of an individual’s apprehension of threat to their bodily or existential integrity [26], consciously-perceived body perception may be viewed as the basis for the appearance of pain.

Researchers have previously developed questionnaires to measure perceptual impairment in complex regional pain syndrome affecting the upper limb [27, 28]; however, so far, no method to evaluate impaired body perception in people with shoulder pain is ready for clinical implementation. The Fremantle Back Awareness Questionnaire (FreBAQ) was developed to evaluate body perception specific to the back in people with chronic low-back pain [29]. The initial development of the FreBAQ was based on the Galer and Jensen [27] complex regional pain syndrome questionnaire, with the addition of items derived from the results of more up-to-date perceptual research [29]. The FreBAQ has since been translated into Japanese [30], Dutch [31], and German [32]. The questionnaire is composed of nine items investigating, neglect-like symptoms, reduced kinesthetic acuity, and perceived body shape and size. The FreBAQ score is related to pain intensity and disability and it has acceptable psychometric properties [29, 30, 33]. A knee-specific body perception questionnaire, the Fremantle Knee Awareness Questionnaire - Japanese (FreKAQ-J), was also developed [34]. The FreKAQ-J has acceptable psychometric properties and is also significantly associated with knee pain intensity and knee pain-related disability [34].

We were interested in investigating whether people with persistent shoulder pain would also endorse symptoms consistent with body perceptual deficits and whether any perceptual impairment relates to clinical status. Changing the key wording of items to allow for use across other musculoskeletal pain issues is one of the methods available to modify a questionnaire, although thorough psychometric testing is essential before adopting the questionnaire into clinical practice. For example, the Keele STarT Back Screening Tool [35–38] and the Fear-Avoidance Beliefs Questionnaire [39–42] for low-back pain were modified for other musculoskeletal disorders including lumbar stenosis and knee, shoulder, and neck pain. Therefore, the aims of this study were to modify the FreBAQ-J by replacing ‘back’ with ‘shoulder’ to enable use for people with shoulder pain, determine whether people with shoulder pain report impairments in self-perception specific to the shoulder, assess the psychometrics of the scale, and investigate whether the scores on this scale relate to clinical status.

## Methods

### Participants

One hundred and twelve participants with persistent shoulder pain were consecutively recruited from an orthopedic outpatient clinic by an orthopedic shoulder surgeon. Participants were included according to the following criteria: (1) age between 20 and 80 years, (2) unilateral shoulder pain for > 3 months, (3) the presence of a positive result for at least three of the following tests: Hawkins impingement sign, Neer’s impingement sign, painful arc sign, Jobe’s test, Whipple’s test, and shoulder pain induced by resisted muscle testing in shoulder abduction or external rotation; and (4) sufficient proficiency in Japanese to complete the questionnaires. All patients underwent an initial radiography examination to screen for fracture, dislocation, degenerative joint disease, and calcific tendinopathy. Participants were excluded according to the following criteria: (1) a history of shoulder surgery or scheduled shoulder surgery, (2) history of shoulder fracture, dislocation, degenerative joint disease of the shoulder, rotator cuff calcific tendinopathy, or complete rotator cuff rupture; (3) active shoulder range of movement < 90 degrees of flexion or abduction or < 0 degrees external rotation, (4) cervical radiculopathy or cervical repeated movement testing affecting shoulder pain and/or range of motion, or (5) other severe orthopedic issue. Forty-eight healthy participants without a history of shoulder pain and matched for age and sex (ratio) with the enrolled patients were recruited as controls. Given the anticipated recruitment challenges, we aimed to recruit in excess of 100 patients to ensure item calibration stability within ±0.5 logits with 95% confidence [43]; this is in keeping with previously established recommendations for minimum sample sizes for Rasch analyses [44]. The calculation for establishing the size of the control group was based on detecting a difference in mean total score between patients and healthy participants. The calculation was undertaken using G*Power 3.1 (University of Kiel, Germany) with a large Cohen effect size, significance set at α = 0.01, and an expected power of (1 − β) = 0.8. This resulted in a sample size of *n* = 39. We aimed to recruit a minimum of 39 healthy participants.

### Development of the Fremantle shoulder awareness questionnaire-Japanese (FreSHAQ-J)

Before changing the FreBAQ we received copyright permission from the developer of the FreBAQ questionnaire. Initially, a Japanese version of the FreBAQ was developed (FreBAQ-J) using a method of forward–backward translation [34]. To develop a shoulder specific version, the Fremantle Shoulder Awareness Questionnaire-Japanese (FreSHAQ-J), the Japanese character for ‘back’ was replaced with the one for ‘shoulder.’ Question 9 in the FreBAQ-J (‘My back feels lopsided’) was considered redundant because the sense of asymmetry does not apply to the shoulder (e.g., the right shoulder alone cannot feel lopsided). This question was adapted to reflect left and right perceptual differences, similar to the knee-specific version [34]. The translation of Question 9 from Japanese to English reads “My shoulders feel different between right and left (in terms of size and shape).”

### Procedure

All participants provided demographic data (age, sex, height, weight). Duration of pain, pain severity, pain-related catastrophizing and shoulder pain-related disability were recorded in participants with shoulder pain since previous studies indicate that these factors are correlated with perceptual impairment [29–34]. Shoulder pain intensity with movement was assessed using a visual analog scale (VAS) anchored at the left with “0 = no pain” and at the right with “100 = unbearable pain.” The level of pain-related catastrophizing was assessed using the Japanese version of the Pain Catastrophizing Scale (PCS) [45, 46]. Functional disability was assessed using the Japanese-validated version of the Quick Disability of the Arm, Shoulder, and Hand (QuickDASH) questionnaire for patients with shoulder pain [47, 48]. All participants also completed the FreSHAQ-J. For those with shoulder pain the instructions used when completing the FreSHAQ-J read ‘please indicate the degree to which your shoulder feels this way when you are experiencing shoulder pain’ and for control participants ‘please indicate the degree to which your shoulder feels this way today.’

### Rasch analysis

We evaluated psychometric properties of the FreSHAQ-J using Rasch analyzes as per the procedures used in previous studies [30, 34]. Rasch analysis enables comparison of ordinal FreSHAQ-J data with probabilistic mathematical models based on the basic principles of measurement [49, 50]. The Rasch model applied here assumes participants with relatively frequent perceptual deficits are more likely to support all of the FreSHAQ-J items and that all participants are more likely to support the items that represent relatively mild perceptual deficits. Rasch analysis was carried out with the program Winsteps (v3.90.2, Chicago, IL) using Andrich rating scale models. The FreSHAQ-J data was analyzed to evaluate the following components.

#### Category order

Rasch analysis facilitates assessment of the FreSHAQ Likert scale, ensuring the categories are used as intended. The FreSHAQ-J has five response categories (0 = Never, 1 = Rarely, 2 = Occasionally, 3 = Often, 4 = Always) and thus four “step calibrations” – the thresholds in which the likelihood of supporting one category is equal to the likelihood of supporting the next category in a properly functioning rating scale. Visual inspection of the Category Probability Curves was conducted to identify category disordering indicative of underutilized categories or misinterpretation of the category labels.

#### Targeting

Targeting refers to the degree to which the FreSHAQ-J items target the participant (persons). Rasch Analysis allows for comparison between the relative item endorsibility and the person agreeability; a scale considered to be well-targeted would one in which the mean item endorsibility, anchored at 0 logits by default, is similar to the mean person agreeability. The distribution of person and item thresholds were analyzed visually and the summary statistics compared to determine how well the items targeted the persons across the range of agreeability. Floor or ceiling effects less than 15% were considered appropriate as indicating by the COSMIN criteria.

#### Internal consistency

Rasch analysis software provides a Person Reliability Index, in additional to the traditional Cronbach’s alpha, as indicators of internal consistency [51], both of which should exceed 0.7 [52].

#### Unidimensionality

Unidimensionality is an assumption of a scale designed to be summed as a measure of a construct. Individual FreSHAQ-J items should contribute to measurement of this unidimensional construct of disturbed body perception but be sufficiently distinct so as not to replicate other items and potentially inflate reliability estimates. Item fit statistics were examined to identify items that deviate from the expected; excessively large fitting residuals (> 1.4 logits) indicate a large difference in the expected and observed performance of the item [53] and that the item assesses constructs other than the intended one. If the fit residuals are too small (< 0.6 logits), the items behave too predictable [53]. We compared both the infit (information-weighed) and outfit (outlier-sensitive) statistics and tested the item-characteristic curves of the misfitting items to determine how they behave against participants of different agreements. As multidimensionality may be too subtle to detected by item fit statistics alone, a Principal Component Analysis (PCA) of residuals was also conducted. Residuals refer to the other activity in the data once the Rasch dimension has been accounted for; if the data fit the model the analysis would expect random noise. Eigenvalues of up to two most likely represent accidental relationships in the data. Eigenvalues larger than two (i.e. the strength of two items) were explored as they could be indicative of a second dimension [54]. Response dependencies between items were investigated by inspecting the residual correlation matrix [55] for pairs of items with correlations greater than 0.4 [56, 57].

#### Person fit

Persons with outfit residuals larger than 1.5 logits were compared with those with outfit statistics using the exact significance test of Fisher [58] (for sex) or the Mann-Whitney U test (for age, pain severity, pain duration, and FreSHAQ-J score). The response strings of missfitting persons were also analyzed to identify whether any response patterns were present.

#### Construct validity

All FreSHAQ-J items share the same Likert scale but contribute differently to measurement of perceptual disturbances. A recent assessment of the FreBAQ [33] found that items differed in terms of endorsability and that the hierarchical ordering of items supported theoretical constructs underlying perceptual disorders, providing evidence of validity [30, 33]. The FreSHAQ-J should function in a manner similar to the original low-back version (FreBAQ J). In the present study, we evaluated the order of items by analyzing the relative endorsability of the items using published FreBAQ-J data [30].

#### Differential item function (DIF)

Items are expected to function in a similar manner for all participants with a similar degree of consistency. DIF analysis identifies individual items that may be biased by factors other than the construct to be measured. DIF was evaluated across five subgroups: sex (males, females), age (<=60, > 60 years), intensity of pain with movement (<=50, > 50 mm, 100 mm VAS, 50 mm), duration of pain (<=12, > 12 months), and disability (median sprit; QuickDASH, <=23, > 23). DIF was tested using the Mantel-Haenszel chi-square test, and the significance was *p* = 0.01 for each item. Item bias was investigated when there was a statistically significant difference, 0.5 logits or greater, between subgroups [59].

### Test-retest reliability

The FreSHAQ-J was re-administered to participants who reported a change less than 10 mm on a 100 mm visual analogue scale measuring pain intensity at two-week follow up. Test-retest reliability was established on the assumption of a relationship between the frequency of pain intensity and perceptual impairments [29, 30, 33]. Intraclass correlation coefficient (ICC) binary mixed models of absolute agreement were calculated. Reliability was classified as poor (ICC3, 1 < 0.40), moderate to good (ICC3, 1 = 0.40–0.75), or excellent (ICC3, 1 = 0.75–1.00) [60].

### Relationship with clinical status

The Statistical Package for Social Sciences Version 22 (IBM SPSS Statistics for Windows, Version 22.0. Armonk, NY: IBM Corp.) was used to evaluate comparisons between patient and control groups and correlational analyses within the patient group. Kolmogorov-Smirnov’s test was used to test for homovariance of data distributions. Student’s t-test was used to compare age, height, weight, and FreSHAQ-J scores between the shoulder pain group and the control group. To verify that sex was not a confounder, we examined sex differences between the shoulder pain group and the control group using Fisher’s exact test. We performed a series of univariate correlations examining the relationships between the FreSHAQ-J total scores and pain intensity, disability, and pain catastrophizing in the shoulder pain group only. Correlations were examined using appropriate parametric or non-parametric tests. The strength of the relationships was interpreted using the recommendations of Cohen [61], namely small (*r* = 0.10), medium (*r* = 0.30), and large (*r* = 0.50). Five separate analyses were performed, and thus the α was adjusted to 0.01.

## Results

Table 1 summarizes the characteristics of the two groups. All participants were native Japanese speakers. Of the shoulder pain participants, 40 (35.7%) were women, the mean age ± SD was 56.2 ± 11.7 years, the mean severity of pain ± SD was 52.9 ± 21.3 and the mean duration of pain ± SD was 12.8 ± 13.4 months. The response frequency for each item of the FreSHAQ-J is listed in Table 2. All nine items were endorsed by patients at some level, though the recorded frequency varied across items. Items 9 had the highest endorsement and Item 7 had the least endorsement. No one endorsed “always” for items 5 and 8.

**Table 1:** Participant characteristics. QuickDASH: Quick Disability of the Arm, Shoulder, and Hand, PCS: Pain Catastrophizing Scale

Characteristics	Shoulder pain (*n* = 112)	Control (*n* = 48)
	Mean (SD) or N (%)	Mean (SD) or N (%)
Demographic information		
Gender (female)	40 (35.7%)	24 (50.0%)
Age (years)	56.2 (11.7)	52.8 (19.9)
Height (cm)	162.0 (8.0)	163.7 (8.5)
Weight (kg)	58.6 (10.8)	61.7 (14.0)
Duration of pain (months)	12.8 (13.4)	
Pain intensity during motion (0–100)	52.9 (21.3)	
Disability (QuickDASH 0–100)	27.8 (19.1)	
Pain Catastrophization (PCS 0–52)	21.1 (10.5)	
FreSHAQ-J (0–36)	9.1 (5.0)	2.5 (3.6)

**Table 2:** Frequency of responses to each item of the FreSHAQ-J

Item	Never N (%)	Rarely N (%)	Occasionally N (%)	Often N (%)	Always N (%)	Median	Mean (SD)
1. My sore shoulder feels as though it is not part of the rest of my body	26 (23.2)	39 (34.8)	31 (27.7)	8 (7.1)	8 (7.1)	1	1.4
2. I need to focus all my attention on my sore shoulder to make it move the way I want it to	42 (37.5)	34 (30.4)	17 (15.2)	13 (11.6)	6 (5.4)	1	1.1
3. I feel as if my sore shoulder sometimes moves involuntarily, without my control	51 (45.5)	33 (29.5)	13 (11.6)	13 (11.6)	2 (1.8)	1	0.9
4. When performing everyday tasks I don’t know how much my sore shoulder is moving	52 (46.4)	27 (24.1)	22 (19.6)	8 (7.1)	3 (2.7)	1	0.9
5. When performing everyday tasks I am not sure exactly what position my sore shoulder is in	52 (46.4)	37 (33.0)	16 (14.3)	7 (6.3)	0 (0)	1	0.8
6. I can’t perceive the exact outline of my sore shoulder	61 (54.5)	27 (24.1)	17 (15.2)	5 (4.5)	2.(1.8)	0	0.7
7. My sore shoulder feels larger than it appears	82 (73.2)	20 (17.9)	5 (4.5)	3 (2.7)	2.(1.8)	0	0.4
8. My sore shoulder feels smaller than it appears	64 (57.1)	33 (29.5)	11 (9.8)	4 (3.6)	0 (0)	0	0.5
9. My shoulders feel different between left and right (in terms of size and shape)	10 (8.9)	23 (20.5)	38 (33.9)	30 (26.8)	11 (9.8)	2	2.0
Total						9	9.1 (5.0)

### Rasch analysis of the FreSHAQ-J

#### Category order

**Fig. 1:**
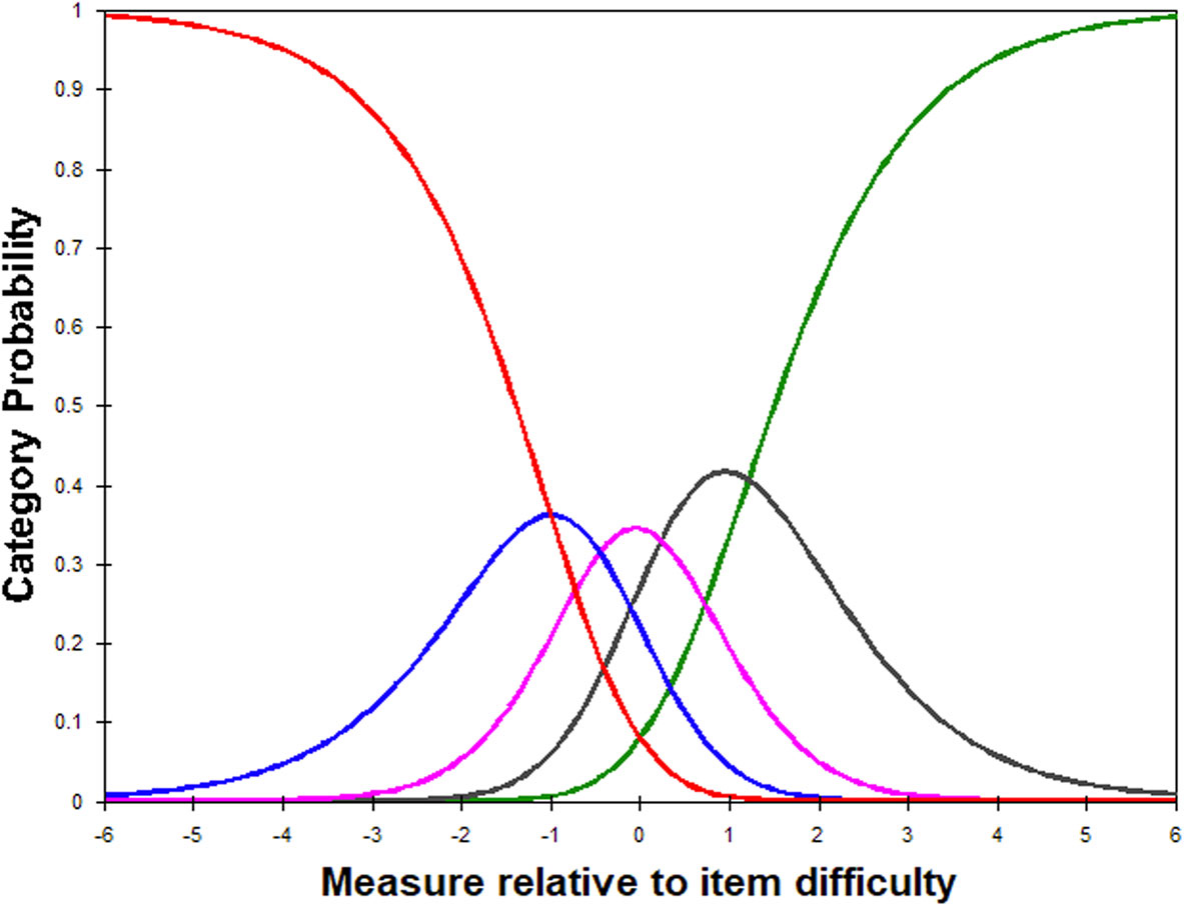
Rasch model category probability curves for the 5-category Japanese version of the Fremantle Shoulder Awareness Questionnaire. (C0, never; C1, rarely; C2, occasionally; C3, sometimes; C4, always)

Visual analysis of the Category Probability Curves demonstrated clear step calibrations suggesting the Likert rating scale functioned as expected (Fig. 1).

#### Targeting

**Fig. 2:**
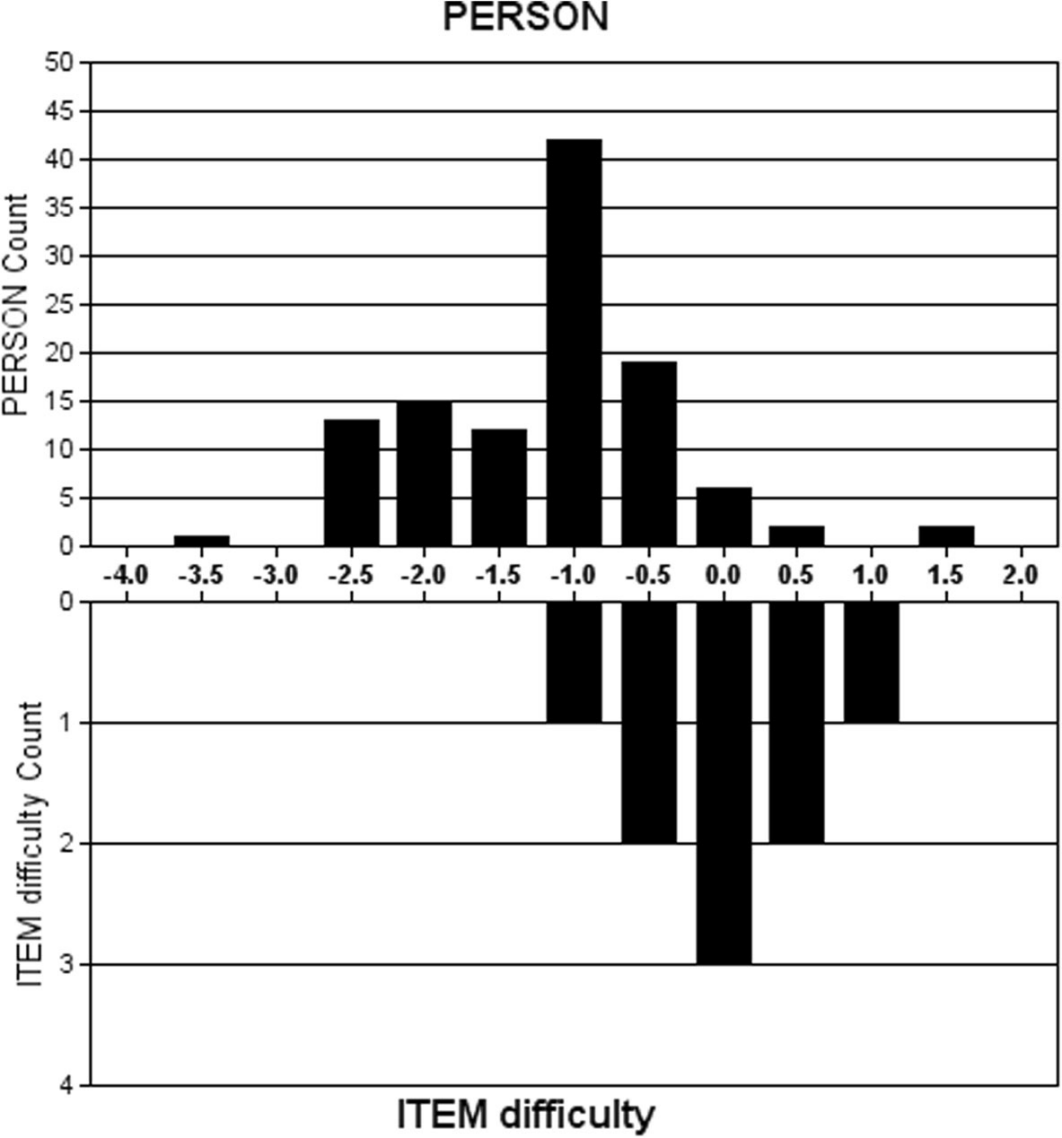
Item-person threshold map. The upper histogram represents the distribution of the person response categories threshold estimates. The lower histogram represents the distribution of the item response categories threshold estimates

Figure 2 shows the association between the FreSHAQ-J items and person logit ratings. Table 3 shows the internal sensitivity thresholds for each item. This sample was not sufficient as a target for the FreSHAQ-J. Persons had a mean agreeability of − 1.21 logits (SD 0.81, range − 3.42 to 1.39), greater than the default item endorsability mean of 0 logits (SD 0.61, range− 1.25 to 0.97). Person agreeability was shifted to the left compared to item endorsibility, indicating that participants with relatively infrequent episodes of body perceptual impairment were not successfully targeted by the scale. Item 9 was endorsed the most by participants. Item 7 was the most difficult to endorse. No participant with shoulder pain scored zero on the questionnaire and none recorded the maximum score.

**Table 3:** Fit statistics from Rasch analysis

Item	Measure (Logits)	SE	Infit (mmsq)	Outfit (mmsq)
9	-1.25	0.10	1.00	1.05
1	−0.54	0.10	1.10	1.04
2	−0.27	0.10	0.99	0.91
4	0.00	0.11	0.86	0.76
3	0.01	0.11	1.12	1.27
5	0.22	0.12	0.77	0.70
6	0.30	0.12	1.06	1.06
8	0.57	0.13	1.28	1.21
7	0.97	0.15	1.13	1.05

#### Internal consistency

The personal reliability index was 0.65, and Cronbach’s alpha was 0.71.

#### Unidimensionality

Table 3 summarizes the fit statistics for the FreSHAQ-J items. No items showed excessive positive and negative infit or outfit. PCA of residuals revealed no clusters of items in the matrix and an eigenvalue of 1.8 units, indicating unidimensionality. No excessive positive or negative correlations (> 0.4) were found between items in the assessment of local dependence.

#### Person fit

Person fit analysis identified 15 (13.3%) participants as having excessive positive outfit (> 1.5 logits). There were no significant differences in age (*p* = 0.85), sex (*p* = 0.56), pain intensity during motion (*p* = 0.41), pain duration (*p* = 0.57), disability (*p* = 0.11), or FreSHAQ-J scores (*p* = 0.35) between patients fitting the Rasch model and those not fitting the model.

#### Construct validity

**Fig. 3:**
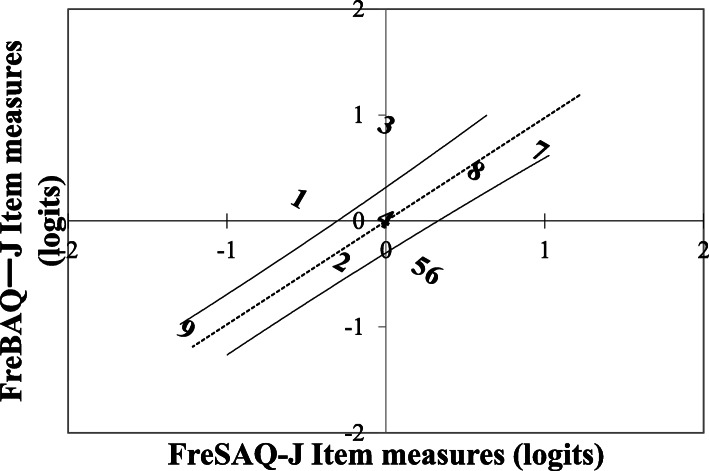
Difference between the FreBAQ-J and FreSHAQ-J. The broken line represents a trend-line through the average of both sets of items, and the solid lines represents the upper and lower 95% confidence bands

The results suggested that the items on the extremities of the scale were stable and that there was some disorder in the intermediate items (Fig. 3). The greatest difference in item function was found for item 3.

#### Differential item function

No items were shown to be significantly biased by any of the assessed variables.

### Test-retest reliability

Thirty participants reported a minimal change in pain intensity and their data were included for analysis. Excellent test-retest reliability was found for the total score, with an ICC_3, 1_ of 0.84 [95% confidence interval (CI) 0.70–0.92].

### Relationship with clinical status

There was no significant difference in age (*p* = 0.24) or sex (*p* = 0.11) between the shoulder pain and control groups. The shoulder pain group scored significantly higher on the FreSHAQ-J than the control group (*p* < 0.001, mean difference = 6.6, 95% CI = 4.9–8.1). The FreSHAQ-J was significantly and moderately correlated with the QuickDASH (rho = 0.38, *p* < 0.01) and PCS (rho = 0.49, *p* < 0.01) (Table 4). However, the FreSHAQ-J did not correlate significantly with pain intensity during movement (rho = 0.20; *p* = 0.03) (Table 4).

**Table 4:** Correlations between the FreSHAQ-J total score and clinical status

	Correlation coefficient (R)	*P* value
Pain intensity during motion	0.20	0.03
Disability (QuickDASH)	0.49	< 0.001
Pain Catastrophization Scale (PCS)	0.38	< 0.001

## Discussion

The aims of the present study were to develop a shoulder-specific body perception questionnaire, to investigate whether people with shoulder pain experience shoulder-specific self-perception deficits, evaluate the psychometric properties of the scale and explore the scales relationship with clinical status. Our results suggested that the newly developed FreSHAQ-J has acceptable categorical order, unidimensionality, no misfitting items, and excellent test-retest reliability. The formation of a unidimensional scale of nine items suggests that the scores can be summed to obtain a measure of perceptual impairment. This scale does not produce bias by age or sex and clinical characteristics such as pain intensity, pain duration, or disability. The category rating scale functions as expected. We observed no floor or ceiling effects in the clinical sample as participants with the lowest or highest possible score were less than 15% of the sample, in fact no participant with shoulder pain recorded either the lowest or highest possible score. Altogether, the FreSHAQ-J showed sufficient psychometric properties to be used for assessing disturbed body perception in people with shoulder pain in a Japanese population.

This is the first study to suggest that people with shoulder pain endorse symptoms consistent with disrupted self-perception and adds some weight to the suggestion that distorted body perception should be targeted as part of the management of people with persistent shoulder pain. How we experience our body is a complex issue with various terms and taxonomies used to try and capture different aspects of body representation. Three main criteria are generally used to distinguish between the different ways the body is viewed and experienced. Firstly, distinction is made between short-term representations and longer-term, more stable representations. Secondly, body representations can be distinguished by their availability to consciousness. Some representations are quite implicit and operate largely outside of consciousness whereas others are unequivocally conscious representations. Lastly, distinction is made about functional role, particularly whether the representation is action orientated or perception orientated [62]. A recent scoping review sought to map and examine the range of literature on functional representations of the body in people with musculoskeletal pain disorders [63]. This group considered the three key domains of body representation pertinent to musculoskeletal pain, the perception of the body (how the body feels to the person), the perception of the space around the body and the sense of ownership of the body, and reviewed both implicit and explicit ways of assessing these domains [63]. Under this taxonomy the FreSHAQ-J would be regarded as measure of explicit body perception, with reasonable face validity as it employs a subjective self-reported scale to measure a subjective consciously felt construct.

The mean availability of the FreSHAQ-J (− 1.21 logits) was consistent with those of the FreBAQ-J (− 0.88 logits) [30] and the FreKAQ-J (− 0.92 logits) [34]. The FreSHAQ-J, similar to the FreBAQ-J, covers moderate and high levels of distortion of body perception and is most suitable for use in people experiencing frequent perceptual dysfunction. In contrast, participants with lower levels of perceptual impairment were not successfully targeted by the FreSHAQ-J. The results suggest that from a psychometric perspective, the addition of new items is needed to assess more subtle impairments. However, it remains to be determined whether low levels of perceptual impairment do in fact impact on clinical status.

The person reliability index was 0.65. This is slightly below the acceptable level of 0.70 and lower than that seen in people with low back pain (FreBAQ-J person reliability index = 0.76) [30] and in people with knee osteoarthritis (FreKAQ-J person reliability index = 0.81) [34]. The person reliability index depends on how targeted the scale is. Low person reliability suggests that questionnaires have limited ability to distinguish distinct classes of perceptual impairment. Sensitivity to change in the questionnaire may also not be ideal. That is, the questionnaire may not be able to detect changes when the patients with high-level body perception disturbance experience improvement in body perception because the FreSHAQ-J does not include questions targeting low-level body perception disturbance. Again, adding scale items that are more easily endorsed and assess lower levels of disturbance should improve the tool. However, Cronbach’s alpha was 0.71, which is higher than the acceptable level of 0.7. There are some differences between Cronbach’s alpha and the person reliability index. Cronbach’s alpha is calculated using all scores, including the maximum and minimum scores, while the estimate of the person reliability index requires extrapolated values for extreme scores. This difference in computation may impact the difference seen between the Cronbach’s alpha and the person reliability index. A minimum value of 0.7 for the Person Reliability Index is recommended for population use and 0.85 for personal use [52]. Therefore, the FreSHAQ-J may be better suited to group use, for example comparing body perception between people with two different conditions, than to use with individual patients.

There were no items showing excessive infit or outfit, and no item bias was observed. Unidimensionality is a key prerequisite for summing a set of items [55, 64, 65]. The present study demonstrated that the scale showed unidimensionality, suggesting that distorted body perception, including three dimensions (neglect-like symptoms, reduced kinesthetic acuity, and perceived body shape and size), was the only construct in people with shoulder pain. Therefore, the FreSHAQ-J allows meaningful comparisons of the degree of body perceptual distortion.

Item hierarchy in the FreSHAQ-J was similar but not identical to that in the FreBAQ-J. The greatest discrepancy between the FreSHAQ J and FreBAQ J was found in item 3 (I feel as if my sore shoulder sometimes moves involuntarily, without my control). Participants with shoulder pain (0.01 logits) endorsed Item 3 to a higher degree than did the participants with low-back pain (0.91 logits). Item 3 was based on symptoms of motor neglect frequently observed in participants with complex regional pain syndrome. Although motor deficits are commonly found in participants with low-back pain [66–68], motor neglect may be observed to a higher degree in participants with limb pain than with low-back pain, and it is certainly plausible that involuntary movement is a more common percept in the limbs than in the trunk.

The ICC of 0.84 (95% CI 0.70–0.92) showed that the FreSHAQ-J has excellent test-retest reliability, confirming the findings of the previous FreBAQ-J study with people with low-back pain (0.81, 95% CI 0.67–0.89) [30] and the FreKAQ-J study assessing people with knee osteoarthritis (0.76, 95% CI 0.52–0.89) [34].

The marked differences in the FreSHAQ-J total scores between the shoulder pain group (mean 9.1) and the control group (mean 2.5) and the relationship with disability and pain catastrophizing, support previous suggestions that perceptual impairment is associated with clinical status in persistent pain [69]. Conversely, unlike the FreBAQ [29, 30, 33] and the FreKAQ [34], this study did not show a relationship between body perceptual disturbance and pain intensity during motion when we corrected for multiple comparisons. Pain intensity in participants with shoulder pain (52.9 ± 21.3 mm) was higher than that in participants with low-back pain (49.1 ± 27.1 mm) [30] and knee osteoarthritis (43.5 ± 24.1 months) [34], whereas the FreSHAQ-J scores in participants with shoulder pain (9.1 ± 5.0) were relatively lower than those in participants with low-back pain (11.7 ± 6.4) or knee osteoarthritis (12.4 ± 7.6). These differences may have influenced the relationship between pain intensity and body perceptual impairment and suggest different impacts of body perceptual dysfunction across different clinical presentations.

Limitations of this study need to be considered. First, the sample size of this study is relatively small. Previous studies suggest that small sample size (*n* < 100) particularly impact on category disorder, targeting, and misfitting items [44]. One hundred and twelve participants contributed data to the Rasch analysis and this may impact on the confidence in some of the results. Some of the issues reported above regarding targeting and misfitting may be less apparent in testing in larger samples. Second, the duration of pain in subjects with shoulder pain (12.8 ± 13.4 months) was shorter than that in subjects with low-back pain (88.8 ± 106.8 months) [30] and knee osteoarthritis (57.7 ± 88.4 months) [34]. Different results to those observed here may be seen in shoulder pain populations with greater chronicity. Third, the sample was derived from an orthopedic outpatient clinic and participants were referred into the study by an orthopedic shoulder surgeon. Further research, particularly including people from primary care, is needed to evaluate the generalizability of the reported findings. Fourth, we did not lodge and lock our protocol and statistical analysis plan prior to data collection. When we commenced this study, such practice was uncommon in our field, but now it is recommended, and is among those at the forefront of this push [70]. Failure to do this clearly represents a shortcoming in transparency and reporting. Lastly, we chose pain intensity as the factor to determine clinical stability for the test-retest part of the study as pain related variables were key inclusion criteria and our previous work has shown consistent relationships between pain intensity and body perception. This relationship was not upheld in this study and an alternate measure such as global perceived effect may be more useful in future investigations.

## Conclusion

The Fremantle Shoulder Awareness Questionnaire, a shoulder-specific body awareness and perception scale was developed by modifying the Fremantle Back Awareness Questionnaire. The FreSHAQ-J fits well to Rasch metric models for people with shoulder pain and has excellent test-retest reliability. The level of disruption in body perception were significantly associated with the clinical status of people with shoulder pain and may be worthwhile assessing when managing this population.

## Data Availability

The datasets used and analyzed during the current study are available from the corresponding author.

## Abbreviations

DIF: Differential item function
FreBAQ: Fremantle Back Awareness Questionnaire
FreKAQ-J: Fremantle Knee Awareness Questionnaire
FreSHAQ-J: Fremantle Shoulder Awareness Questionnaire-Japanese
ICC: Intraclass correlation coefficient
PCA: Principal component analysis
PCS: Pain Catastrophizing Scale
QuichDASH: Quick Disability of the Arm, Shoulder, and Hand
VAS: Visual analog scale
